# Triglyceride (Palmkernöl): MAK-Begründung, Nachtrag

**DOI:** 10.34865/mbtriglykskd10_2ad

**Published:** 2025-06-30

**Authors:** Andrea Hartwig, MAK Commission

**Affiliations:** 1 Institut für Angewandte Biowissenschaften. Abteilung Lebensmittelchemie und Toxikologie. Karlsruher Institut für Technologie (KIT) Adenauerring 20a, Geb. 50.41 76131 Karlsruhe Deutschland; 2 Ständige Senatskommission zur Prüfung gesundheitsschädlicher Arbeitsstoffe. Deutsche Forschungsgemeinschaft, Kennedyallee 40, 53175 Bonn, Deutschland. Weitere Informationen: Ständige Senatskommission zur Prüfung gesundheitsschädlicher Arbeitsstoffe | DFG

**Keywords:** Palmkernöl, Lungentoxizität, Überladungseffekt, Entzündung, Mikrogranulom, MAK-Wert, maximale Arbeitsplatzkonzentration, Aerosol, Entwicklungstoxizität, Analogiebetrachtung, palm kernel oil, lung toxicity, overload effect, inflammation, microgranuloma, MAK value, maximum workplace concentration, aerosol, developmental toxicity, read-across

## Abstract

The German Senate Commission for the Investigation of Health Hazards of Chemical Compounds in the Work Area (MAK Commission) evaluated the data for palm kernel oil [8023-79-8] to derive an occupational exposure limit value (maximum concentration at the workplace, MAK value) considering all toxicological end points. Relevant studies were identified from a literature search. Palm kernel oil is an oily extract of plant origin made up of a mixture of fatty acid triglycerides and thus a UVCB substance (substance of Unknown or Variable composition, Complex reaction products or Biological materials). As in the case of white mineral oil, inhalation of the poorly water-soluble palm kernel oil in aerosol form may result in lung overload, inflammatory reactions and microgranulomas. To prevent these effects of overloading, a MAK value of 5 mg/m^3^ has been derived for the respirable fraction as a worst-case assumption in analogy to the value established for white mineral oil. The amount of palm kernel oil taken up after inhalation exposure at the MAK value of 5 mg/m^3^ is several orders of magnitude lower than the level of uptake with the diet and remains below the recommended daily dietary intake for fats. Peak Limitation Category II with an excursion factor of 4 has been set. No prenatal developmental studies of palm kernel oil were carried out in compliance with valid test guidelines. The induction of teratogenic effects is unlikely due to the structure of the fatty acids that form from the oil. Secondary effects on the foetus due to maternal hypoxia caused by a lung overload are not expected if the MAK value of 5 mg/m^3^ is observed. Therefore, palm kernel oil has been assigned to Pregnancy Risk Group C. There is no indication of a genotoxic or carcinogenic potential of palm kernel oil. No data for sensitization are available. Skin contact is not expected to contribute significantly to systemic toxicity.

**Table:** 

**MAK-Wert (2024)**	**5 mg/m^3^ A**
**Spitzenbegrenzung (2024)**	**Kategorie II, Überschreitungsfaktor 4**
	
**Hautresorption**	**–**
**Sensibilisierende Wirkung**	**–**
**Krebserzeugende Wirkung**	**–**
**Fruchtschädigende Wirkung (2024)**	**Gruppe C**
**Keimzellmutagene Wirkung**	**–**
	
**BAT-Wert**	**–**
	
Synonyma	Palmnussöl
Chemische Bezeichnung (IUPAC-Name)	–
CAS-Nr.	8023-79-8
Formel	–
Molmasse	–
Schmelzpunkt	25–30 °C (Burnett et al. 2017; Cornelius 1977), 26–30 °C (Johnson 2000)
Siedepunkt	203 °C (NCBI 2015)
Dichte bei 15 °C	0,925–0,935 g/cm^3^ (NCBI 2015)
Dampfdruck	k. A.
log K_OW_	k. A.
Löslichkeit	unlöslich in Wasser (Johnson 2000)
Hydrolysestabilität	Verseifungszahl (mg Kaliumhydroxid/g Fett): 245–255 (Burnett et al. 2017), 242–254 (DGF o.J.), 244–254 (Cornelius 1977)
Stabilität	Bei Luftkontakt kann Palmkernöl oxidieren und ranzig werden.
Herstellung	Palmkernöl wird aus den Samen der Ölpalme (Elaeis guineensis) gewonnen, deren Früchte zur Gewinnung von Palmöl verwendet werden (Johnson 2000). Die Ölextraktion erfolgt durch Pressung oder mittels Lösemittel (Cornelius 1977)
Reinheit	k. A.
Verunreinigungen	k. A.
Verwendung	Zur Herstellung von Detergenzien, Pharmazeutika, Malstiften, Kerzen und Weißblech (Burnett et al. 2017), Inhaltsstoff von Kosmetika (Johnson 2000), hochwertigen Seifen und Formulierungen für Margarine, Backfett und Kochöle (Cornelius 1977)
maximale Einsatzkonzentration	50 % im Kühlschmierstoffkonzentrat (Hartwig und MAK Commission 2023)

Palmkernöl ist ein Gemisch verschiedener Triglyceride. Im Folgenden wird der Begriff „Triglyceride“ dahingehend verstanden, dass es sich um aus Pflanzen und Tieren gewonnene Öle auf Fettsäuretriglyceridbasis handelt. Hauptsächlich besteht Palmkernöl aus Triglyceriden mit Laurinsäure (12:0 (Anzahl der Kohlenstoffatome:Anzahl der Doppelbindungen); 44,5–52,0 %), Myristinsäure (14:0; 14,0–18,6 %), Ölsäure (18:1; 10,5–18,5 %), Palmitinsäure (16:0; 6,5–10,4 %), Caprylsäure (8:0; 2,4–4,3 %) und Caprinsäure (10:0; 3,0–6,3 %). Weitere Fettsäuren haben jeweils einen maximalen Gewichtsanteil von unter 4 % (Cornelius 1977). Es handelt sich also um einen UVCB-Stoff (Substanzen unbekannter oder variabler Zusammensetzung, komplexe Reaktionsprodukte oder biologische Materialien).

Es liegen bereits Begründungen zu den Triglyceriden Lardöl, Palmöl, Rapsöl und Sojaöl (Hartwig und MAK Commission 2022) sowie zu Kokosnussöl (Hartwig und MAK Commission 2019) vor.

Für Palmkernöl gilt wie für andere Triglyceride, einschließlich Kokosnussöl, dass es sich um ein eher zähflüssiges Öl mit niedrigem Dampfdruck handelt, sodass eine mögliche Exposition vorwiegend als Aerosol erfolgt. Diese Öle sind nicht oder allenfalls gering reizend an Haut und Auge und wirken nach oraler Aufnahme erst ab mehreren Gramm pro kg Körpergewicht systemisch toxisch (Hartwig und MAK Commission 2019, 2022).

## 1 Allgemeiner Wirkungscharakter

Bei oraler Gabe von Palmkernöl im Gramm/kg-KG-Bereich kommt es im Tierversuch zu Veränderungen, die für eine vermehrte Fettzufuhr typisch sind. So verursacht Palmkernöl bei Ratten erhöhte Gesamt-Cholesterin-Werte im Plasma und in den Nieren. Bei Kaninchen treten in diesem Dosisbereich erhöhte Gesamt-Cholesterin-Werte in Serum und Leber sowie histopathologische Veränderungen in Leber, Lunge und Aorta auf.

Eine Inhalationsstudie mit Palmkernöl liegt nicht vor. Bei Aerosol-Exposition gegen Palmkernöl ist in Analogie zu Weißöl mit einer Makrophagenansammlung und der Bildung von Mikrogranulomen in der Lunge zu rechnen.

Palmkernöl wirkt leicht reizend an der Kaninchenhaut.

Zur sensibilisierenden Wirkung von Palmkernöl liegen keine Daten vor.

Hinweise auf eine fertilitätsschädigende oder entwicklungstoxische Wirkung gibt es für Palmkernöl nicht.

Ein genotoxisches oder kanzerogenes Potenzial ist für Palmkernöl nicht gegeben.

## 2 Wirkungsmechanismus

Palmkernöl ist wie alle Fettsäuretriglyceride im Organismus hydrolysierbar. Bei Überschreitung der Hydrolyse-Kapazität kann es in der Lunge zu einem Überladungseffekt kommen. Die Grenze der Hydrolyse-Kapazität ist nicht bekannt, d. h. es ist unklar, bei welcher Konzentration mit einer Überladung in der Lunge zu rechnen ist. Dieser Überladungseffekt führt zu entzündlichen Reaktionen und Mikrogranulomen bis hin zu fibrotischen Veränderungen und ist für Weißöl, einem nicht hydrolysierbaren Mineralöl, im Tiermodell experimentell belegt (Hartwig 2015). Der Wirkungsmechanismus ist in der Begründung zu den Triglyceriden dargestellt (Hartwig und MAK Commission 2022).

Gesättigte pflanzliche Öle wie Kokosnuss-, Palm- und Palmkernöl enthalten vorwiegend gesättigte Fettsäuren (Laurin-, Myristin- und Palmitinsäure). Diese führen vermutlich über eine reduzierte LDL (Low Density Lipoprotein)-Rezeptor-Aktivität oder eine gesteigerte LDL-C (LDL-Cholesterin)-Produktion zur Erhöhung der Gesamtcholesterinwerte und der LDL-C-Werte im Blut von Mensch und Tier (NCBI 2015).

## 3 Toxikokinetik und Metabolismus

### 3.1 Aufnahme, Verteilung, Ausscheidung

Daten zur inhalativen und dermalen Aufnahme liegen weder für Palmkernöl noch für andere Triglyceride vor.

Nach oraler Aufnahme werden im Duodenum Triglyceride durch die Pankreaslipase in Fettsäuren, Glycerin und Mono- bzw. Diglyceride gespalten und in die Enterozyten aufgenommen. Im Allgemeinen wird angenommen, dass die Kettenlänge der Fettsäuren die orale Resorption bestimmt. Während freie Fettsäuren ab einer Kettenlänge von 14 Kohlenstoffatomen (Öl-, Linol-, Linolen-, Palmitin-, Gadolein-, Stearin-, Myristin-, Palmitoleinsäure) in den Enterozyten wieder zu Triglyceriden verestert werden, gelangen freie Fettsäuren der Kettenlänge von 8 bis 12 Kohlenstoffatomen direkt über die Pfortader zur Leber. Im Serum werden die Triglyceride an Lipoproteine gebunden oder als Chylomikronen über das lymphatische System transportiert und im Fettgewebe gespeichert. Freie Fettsäuren, die aus dem Fettgewebe wieder freigesetzt werden, werden entweder an Serumalbumine gebunden oder verbleiben als nicht veresterte Fettsäuren im Blut. Die physiologische Konzentration von freien Fettsäuren im Blutplasma beträgt 10 bis 300 mg/l. Die enterale Resorption freier Fettsäuren nimmt mit zunehmender Kettenlänge ab (Hartwig und MAK Commission 2022).

Bei sechs männlichen adulten Wistar-Ratten pro Gruppe wurde die Aufnahme von Palmkernöl und Maiskeimöl, die jeweils [1,2-^3^H]-Cholesterin enthielten, untersucht. Dazu wurden die Tiere anästhesiert und im Duodenum und in den thorakalen Lymphgängen Katheter gelegt. Über den Katheter im Duodenum wurden die Öle infundiert und 24 Stunden lang über den anderen Katheter Lymphflüssigkeit gesammelt. Sechs Kontrolltiere erhielten eine lipidfreie Emulsion. Im Vergleich zu den Kontrolltieren war in der Lymphflüssigkeit der Anteil der Laurinsäure um 13,5 % und der Myristinsäure um 7,3 % höher. Im Vergleich zu Maiskeimöl, für das eine 100%ige Aufnahme angenommen wurde, lag die Aufnahme von Palmkernöl bei 82 % (Chen et al. 1989).

### 3.2 Metabolismus

Nach oraler Aufnahme werden im Duodenum Triglyceride durch die Pankreaslipase in Fettsäuren, Glycerin und Mono- bzw. Diacylglyceride gespalten. Der Abbau der Fettsäuren erfolgt im Fettstoffwechsel über sukzessive β-Oxidationen der jeweils endständigen C2-Einheit als Essigsäurethioester des Coenzyms A. Der Abbau der Fettsäuren kann in untergeordnetem Maße in der Leber auch durch ω-Oxidation und im Gehirn durch α-Oxidation erfolgen. Die Fettsäuren sind in Form ihrer Triglyceride natürliche Bestandteile pflanzlicher und tierischer Fette (Neutralfette) und unterliegen dem allgemeinen Fettsäure-Stoffwechsel (Hartwig und MAK Commission 2022).

## 4 Erfahrungen beim Menschen

### 4.1 Einmalige Exposition

Hierzu liegen keine Angaben vor.

### 4.2 Wiederholte Exposition

Über die Nahrung aufgenommene gesättigte Fettsäuren führten verglichen mit ein- und mehrfach ungesättigten Fettsäuren zu erhöhten Cholesterinwerten (LDL-Cholesterin und/oder Gesamtcholesterin über den Referenzbereich hinaus). Von den kommerziell verfügbaren Ölen bewirkten Palmkernöl und Kokosnussöl, die den höchsten Anteil an gesättigten Fettsäuren aufweisen, dies am effektivsten (NCBI 2015). Erhöhte LDL-Cholesterinwerte gelten als Risikofaktor für kardiovaskuläre Erkrankungen wie Herzinfarkt oder Schlaganfall (EFSA Panel on Dietetic Products, Nutrition, and All﻿ergies (NDA) 2010).

### 4.3 Wirkung auf Haut und Schleimhäute

In klinischen Studien wirkten 15 % Palmkernöl in Vaseline oder kosmetische Formulierungen mit 1,0 bis 2,0 % Palmkernöl nicht reizend (k. w. A.; Burnett et al. 2017). Flocken von Seifenstücken zeigten sich in verdünnten Lösungen bis 2,13 % Palmkernöl, der höchsten getesteten Konzentration, als nicht reizend (k. w. A.; Johnson 2000).

### 4.4 Allergene Wirkung

#### 4.4.1 Hautsensibilisierende Wirkung 

In einem Repeated Insult Patch Test (RIPT) mit Flocken von Seifenstücken in verdünnten Lösungen mit maximal 2,13 % Palmkernöl zeigten zwei von 119 Probanden schwache Erytheme während der Induktion. Ein weiterer Proband wies während Induktion und Auslösung ebenfalls schwache Erytheme auf. Eine kontaktsensibilisierende Wirkung wurde nicht beobachtet (Johnson 2000). Wegen der geringen eingesetzten Substanzkonzentration ist dieses Ergebnis jedoch nicht für die Bewertung heranziehbar. Die unveröffentlichte Originalstudie liegt nicht vor.

Es gibt ferner Ergebnisse aus einem RIPT mit einem Badegel, das 1 % ethoxyliertes Palmkernöl enthielt, die von den Studienautoren als klinisch nicht relevant bewertet wurden (Johnson 2000). Diese Studie wird ebenfalls aufgrund der geringen eingesetzten Substanzkonzentration und der Modifizierung des Palmkernöls nicht für die Bewertung herangezogen.

Für ähnliche Triglyceride (Lard-, Palm-, Raps-, Soja-, Kokosnussöl) liegen keine Hinweise auf eine sensibilisierende Wirkung vor (Hartwig und MAK Commission 2019, 2022).

#### 4.4.2 Atemwegssensibilisierende Wirkung 

Hierzu liegen keine Angaben vor.

### 4.5 Reproduktionstoxizität

Hierzu liegen keine Angaben vor.

### 4.6 Genotoxizität

Hierzu liegen keine Angaben vor.

### 4.7 Kanzerogenität

Es gibt zahlreiche epidemiologische Studien zum Zusammenhang zwischen erhöhter Fett- bzw. Fettsäureaufnahme mit der Nahrung und dem Auftreten von Tumoren. Da die Vorstellungen über den zugrundeliegenden Mechanismus uneinheitlich sind und zudem eine Relevanz für die Expositionssituation am Arbeitsplatz aufgrund der um ein Vielfaches geringeren Aufnahme fehlt, werden diese Studien nicht für die Bewertung der Kanzerogenität herangezogen (Hartwig und MAK Commission 2022).

## 5 Tierexperimentelle Befunde und In-vitro-Untersuchungen

### 5.1 Akute Toxizität

#### 5.1.1 Inhalative Aufnahme

Hierzu liegen keine Angaben vor.

#### 5.1.2 Orale Aufnahme

Die orale LD_50_ für Ratten wird mit mehr als 5000 mg/kg KG angegeben (k. w. A.; NCBI 2015; OECD 2014).

#### 5.1.3 Dermale Aufnahme

Hierzu liegen keine Angaben vor.

### 5.2 Subakute, subchronische und chronische Toxizität

#### 5.2.1 Inhalative Aufnahme

Hierzu liegen keine Angaben vor.

#### 5.2.2 Orale Aufnahme

Die Studien zur wiederholten oralen Gabe von Palmkernöl sind in Tabelle 1 dargestellt. Studien nach gültigen Prüfrichtlinien liegen für Palmkernöl nicht vor.

Bei drei Wochen alten, weiblichen Cpb:WU-Ratten führte die 21-tägige Gabe von 15 Gew.-% (Gewichtsprozent) Palmkernöl im Futter (ca. 18 000 mg/kg KG und Tag; Umrechnungsfaktor 0,12 subakut nach EFSA Scientific Committee (2012)) im Vergleich zu einer Gruppe, die Sojaöl erhielt, zu erhöhten Gesamtcholesterinwerten im Plasma (Zhang et al. 1990).

Bei männlichen Ratten, die zwölf Wochen lang 5 Gew.-% Palmkernöl im Futter (ca. 5170 mg/kg KG und Tag; Umrechnungsfaktor 0,09 subchronisch nach EFSA Scientific Committee (2012)) erhalten hatten, kam es im Vergleich zur Kontrollgruppe (unbehandeltes Futter) in den Nieren zu erhöhten Gesamtcholesteringehalten. Gleichzeitig gab es keine Effek﻿te auf die Konzentrationen von Gesamtcholesterin, HDL-C, LDL-C und Triglyceriden (Ebesunun et al. 2003).

Männliche Neuseeländer-Kaninchen, die neun Monate lang 14 Gew.-% Palmkernöl im Futter (ca. 4200 mg/kg KG und Tag; unter der Annahme eines KG von 2 kg und einer täglichen Futteraufnahme von 60 g; Nielsen et al. 2008, S. 338) erhalten hatten, wiesen im Vergleich zur Kontrollgruppe (Maiskeimöl) erhöhte Gesamtcholesterinwerte in Serum und Leber sowie Leberhypertrophie auf (Kritchevsky et al. 1982).

Weiblichen Holländer-Kaninchen wurden bei einer Untersuchung zur Entstehung der Atherosklerose 54 Wochen lang 40 % der Kalorien im Futter in Form von Palmkernöl (k. A. zu Gew.-% im Futter; Annahme 20 Gew.-% Palmkernöl im Futter, 2 kg KG, Futteraufnahme 60 g/Tag (Nielsen et al. 2008, S. 338): ca. 6000 mg/kg KG und Tag) verabreicht. Im Vergleich zur Kontrollgruppe (Sonnenblumenöl) kam es zu erhöhten Gesamtcholesterinwerten im Serum und zu histologischen Veränderungen in Leber (fettige Infiltrationen), Lunge (atherosklerotische Arterien, Cholesteringranulome) und Aorta (Atherosklerose) (Kloeze und Abdellatif 1975).

**Tab. 1:** Toxizität von Palmkernöl nach wiederholter oraler Verabreichung

**Spezies, ** **Stamm, ** **Anzahl pro Gruppe**	**Exposition**	**Befunde**	**Literatur**
Ratte, Cpb:WU, 12 ♀, Alter: 3 Wo	21 d, 15 Gew.-% im Futter (ca. 18 000 mg/kg KG u. d^a)^), Vergleichsgruppen: Kokosnussfett, Palmöl, Sojaöl, Rapsöl: je 15 Gew.-% im Futter, 7 d/Wo	**ca. 18 000 mg/kg KG**: Plasma: Gesamtcholesterin ↑ im Vgl. zur Sojaöl-Gruppe (Gruppe mit den niedrigsten Werten); kein Effekt: KG, Lebergewicht	Zhang et al. 1990
Ratte, Albino (k. w. A.), 6 ♂	12 Wo, 5 Gew.-% im Futter (ca. 5170 mg/kg KG u. d^b)^), Kontrollgruppe: unbehandeltes Futter, 7 d/Wo	**ca. 5170 mg/kg KG**: Nieren: Gesamtcholesterin ↑; kein Effekt: Plasma: Gesamtcholesterin, HDL-C, LDL-C, Triglyceride	Ebesunun et al. 2003
Kaninchen, Neuseeländer, 8 ♂, Kontrollgruppe: 12 ♂	9 Mo, 14 Gew.-% im Futter (ca. 4200 mg/kg KG u. d^c)^), Kontrollgruppe: Maiskeimöl: 14 Gew.-% im Futter, 7 d/Wo	**ca. 4200 mg/kg KG**: Serum: Gesamtcholesterin ↑ (436 mg/dl, Kontrolle: 64 mg/dl), Leber: Gesamtcholesterin ↑, Hypertrophie	Kritchevsky et al. 1982
Kaninchen, Holländer, 12 ♀	54 Wo, 40 Kalorien-% im Futter (k. A. zu Gew.-% im Futter; Annahme 20 %: ca. 6000 mg/kg KG u. d^c)^), Kontrollgruppe: Sonnenblumenöl 40 Kalorien-% im Futter, 7 d/Wo	**ca. 6000 mg/kg KG**: Serum: Gesamtcholesterin ↑, Leber: fettige Infiltrationen (3/10, Kontrolle: 1/9), Lunge: atherosklerotische Arterien (1/10, Kontrolle: 0/9) u. Cholesteringranulome (1/10, Kontrolle: 0/9), Aorta: Atherosklerose Schweregrad ↑	Kloeze und Abdellatif 1975

^a)^Umrechnungsfaktor 0,12 subakut nach EFSA Scientific Committee (2012)

^b)^Umrechnungsfaktor 0,09 subchronisch nach EFSA Scientific Committee (2012)

^c)^unter der Annahme eines KG von 2 kg und einer täglichen Futteraufnahme von 60 g (Nielsen et al. 2008, S. 338)

**Fazit:** Effekte sind mit Palmkernöl wie auch mit den Triglyceriden Lardöl, Palmöl, Rapsöl, Sojaöl und Kokosnussöl (Hartwig und MAK Commission 2019, 2022) erst im Gramm/kg-KG-Bereich zu sehen. Die Struktur von Palmkernöl gibt keinen Verdacht auf eine hohe systemische Toxizität.

#### 5.2.3 Dermale Aufnahme

Hierzu liegen keine Angaben vor.

### 5.3 Wirkung auf Haut und Schleimhäute

#### 5.3.1 Haut

An Kaninchen wurde das hautreizende Potenzial von unverdünntem Palmkernöl untersucht. Dazu wurden 0,5 ml der Testsubstanz unter semiokklusiven Bedingungen vier Stunden lang auf die geschorene Rückenhaut aufgebracht. Direkt nach Expositionsende sowie 24, 48 und 72 Stunden danach wurde die Haut untersucht. Überwiegend zeigte die Testsubstanz leichte Effekte; zwei bis drei Hautstellen wiesen 24 und 72 Stunden nach der Behandlung eine leichte bis mäßige Reizung auf. Die Testsubstanz wurde als leicht reizend bewertet (k. w. A.; ECB 2000).

Im Rahmen einer Kategorie-Bewertung von Glyceriden wurde Palmkernöl als nicht reizend bis leicht reizend für die Kaninchenhaut beschrieben (k. w. A.; OECD 2014).

Die Triglyceride Lardöl, Palmöl, Rapsöl, Sojaöl und Kokosnussöl wirken nicht bis allenfalls minimal reizend an der Haut (Hartwig und MAK Commission 2019, 2022).

#### 5.3.2 Auge

Hierzu liegen keine Angaben vor.

Die Triglyceride Lardöl, Palmöl, Rapsöl, Sojaöl und Kokosnussöl wirken nicht bis allenfalls minimal reizend am Auge (Hartwig und MAK Commission 2019, 2022).

### 5.4 Allergene Wirkung

Hierzu liegen keine Angaben vor.

### 5.5 Reproduktionstoxizität

Adulte mongolische Wüstenrennmäuse (hochresistent gegen Atherosklerose) und ihre Nachkommen (erste Generati﻿on, fünf Paare/Gruppe) erhielten unbehandeltes Futter oder 8,75 Gew.-% Palmkernöl im Futter. In der zweiten Generati﻿on wurden bei den mit Palmkernöl gefütterten Tieren keine statistisch signifikanten Unterschiede im Vergleich zur Kontrollgruppe bei der Wurfhäufigkeit, der durchschnittlichen Wurfgröße, der Gesamtzahl der Neugeborenen und der Anzahl von Todesfällen unter den gesäugten Jungtieren festgestellt (k. w. A.; Johnson 2000).

Palmkernöl zeigte in OECD-Prüfrichtlinie-416-konformen Studien keine Effekte auf die Fertilität oder die Entwicklungstoxizität von Ratten, Mäusen oder Hamstern (k. w. A.; OECD 2014).

Für die Triglyceride Lardöl, Palmöl, Rapsöl, Sojaöl und Kokosnussöl gibt es keine Hinweise auf eine fertilitätsschädigende oder entwicklungstoxische Wirkung (Hartwig und MAK Commission 2019, 2022).

### 5.6 Genotoxizität

Hierzu liegen keine Angaben vor.

Die Triglyceride Lardöl, Palmöl, Rapsöl, Sojaöl und Kokosnussöl zeigen kein genotoxisches Potenzial (Hartwig und MAK Commission 2019, 2022).

### 5.7 Kanzerogenität

Kanzerogenitätsstudien sind mit Palmkernöl nicht durchgeführt worden.

Bei weiblichen Sprague-Dawley-Ratten wurde in einem Initiations-Promotions-Experiment der Effekt von Palmkernöl auf die 7,12-Dimethylbenz[a]anthracen (DMBA)-induzierte Tumorentstehung in der Brustdrüse untersucht. Die Tie﻿re erhielten im Alter von 50 Tagen eine einzelne orale Dosis DMBA (5 mg/0,5 ml Maiskeimöl) und beginnend zwei Tage danach 15 Wochen lang Futter mit verschiedenen Ölen (20 %; ca. 18 000 mg/kg KG und Tag, Umrechnungsfaktor 0,09 (subchronisch) nach EFSA Scientific Committee (2012)). Die verabreichten Öle waren Maiskeimöl, Kakaobutter, Erdnussöl, Palmkernöl oder Butterfett. Eine Kontrollgruppe gab es nicht. Am Studienende lag die Anzahl der Tumoren (tastbare und nicht tastbare) bei den Palmkernöl-gefütterten Tieren bei 57. Am höchsten war die Anzahl bei den Maiskeimöl-behandelten Tieren (78 Tumoren) und am niedrigsten bei den Kakaobutter-behandelten Tieren (45 Tumoren). Die Tumorlatenzzeit war in allen Gruppen ähnlich hoch. Auch gab es keine Unterschiede bei der Anzahl tastbarer Tumoren pro Tumor-tragender Ratte, Gesamtzahl von Tumoren pro Tumor-tragender Ratte, Tumorgewicht und Anzahl der Tumoren pro Tier zwischen den Behandlungsgruppen. Die Anzahl tastbarer Tumoren und die Gesamtzahl von Tumoren bezogen auf die Behandlungsgruppe korrelierte mit dem Anteil von mehrfach ungesättigten Fettsäuren und dem Verhältnis von mehrfach ungesättigten zu gesättigten Fettsäuren im Futter (k. w. A.; Johnson 2000). In dieser Veröffentlichung wird auf die Zusammenfassung einer Tagung Bezug genommen. Da keine Kontrollgruppe verwendet wurde und die Expositionsdauer mit 15 Wochen zu kurz war, kann mit dieser Studie die kanzerogene Wirkung von Palmkernöl nicht bewertet werden.

Ein kanzerogenes Potenzial ist für die Triglyceride Lardöl, Palmöl, Rapsöl, Sojaöl und Kokosnussöl nicht gegeben (Hart﻿wig und MAK Commission 2019, 2022).

## 6 Bewertung

Kritischer Effekt ist eine mögliche Lungentoxizität, wie sie von Weißöl bekannt ist.

**MAK-Wert. **Da Palmkernöl ähnlich wie die Triglyceride Lardöl, Palmöl, Rapsöl, Sojaöl und Kokosnussöl ein eher zähflüssiges Öl mit niedrigem Dampfdruck ist, ist keine Dampf-, sondern nur eine Aerosol-Exposition zu erwarten. Diese könnte wie bei Weißöl in der Lunge zu einer Makrophagenansammlung und zur Bildung von Mikrogranulomen führen. Mit Weißöl kann es bei wiederholter inhalativer Exposition zu einem Überladungseffekt in der Lunge kommen (Hartwig 2015). Während Mineralöle nicht hydrolysierbar sind, können Triglyceride in den Alveolarmakrophagen jedoch der Hydrolyse unterliegen. Bei Überschreitung der Hydrolysekapazität kann es aber auch mit diesen Substanzen zu einer Überladung in der Lunge kommen. Die Grenze der Hydrolysekapazität ist nicht bekannt, d. h. es ist unklar, bei welcher Konzentration mit einem Überladungseffekt zu rechnen ist.

Für Palmkernöl wird, wie für andere Triglyceride auch (Hartwig und MAK Commission 2019, 2022), ein vorläufiger MAK-Wert von 5 mg/m^3^ A in Analogie zu Weißöl festgesetzt. Pflanzliche und tierische Triglyceride führen in der Lunge zu ähnlichen, jedoch weniger starken, Effekten wie Mineralöl. Der festgesetzte MAK-Wert von 5 mg/m^3^ A ist daher ein konservativer Ansatz. Diese Konzentration wird bei Exposition am Kühlschmierstoffarbeitsplatz bei Einhaltung des entsprechenden technisch basierten Grenzwerts von 10 mg/m^3^ E nicht erreicht. Die zu erwartende inhalative und dermale Aufnahme von Palmkernöl am Arbeitsplatz ist um mehrere Größenordnungen niedriger als eine Aufnahme mit der Nahrung. Bei Einhaltung des technisch basierten Grenzwertes für Kühlschmierstoffe von 10 mg/m^3^ E lässt sich für Arbeitsplätze mit Kühlschmierstoffexposition eine Aufnahme von 100 mg (10 m^3^/8 Stunden, 100 % Resorption), entsprechend 1,4 mg/kg KG und Tag bei einem Körpergewicht von 70 kg berechnen. Auch wenn der Kühlschmierstoff nur aus Palmkernöl bestehen würde, wäre die inhalative Aufnahme nur im mg/kg-KG-Bereich. Die empfohlene Aufnahme von Fetten mit der Nahrung liegt dagegen im Gramm/kg-KG-Bereich (DGE 2015).

**Spitzenbegrenzung. **Bei Lungeneffekten durch Überladung handelt es sich um eine kumulative, spät eintretende Wirkung (Hartwig 2015), sodass die Zuordnung zu Spitzenbegrenzungs-Kategorie II erfolgt. Analog zum pharmazeutischen Weißöl bzw. den Polyalphaolefinen wird ein Überschreitungsfaktor von 4 zur Spitzenbegrenzung festgesetzt, da kurzzeitig sehr hohe Konzentrationen möglicherweise das Verteilungsverhalten in den Alveolen und damit auch die Verweilzeit ändern könnten. Die Bildung von Mikrogranulomen in der Lunge muss verhindert werden (Hartwig 2011).

**Fruchtschädigende Wirkung. **Entwicklungstoxizitätsstudien liegen zu Palmkernöl nicht vor.

Palmkernöl kann aufgrund der Zusammensetzung wie die anderen Triglyceride bewertet werden. In der Begründung zu den Triglyceriden ist ausführlich dargelegt, dass bei Exposition in Höhe des MAK-Wertes von 5 mg/m^3^ keine entwicklungstoxischen Effekte zu erwarten sind. Als Argumente kommen zum Tragen, dass diese Hauptbestandteile der Nahrung des Menschen sind, der Abstand zwischen dem auf eine Körperdosis umgerechneten MAK-Wert und der empfohlenen Gesamtfett-Aufnahme mehrere Größenordnungen beträgt, kein entsprechender Strukturverdacht für die Triglyceride und ihre Abbauprodukte besteht und Sekundäreffekte auf den Fetus durch eine mögliche Hypoxie durch den Überladungseffekt in der mütterlichen Lunge ausgeschlossen sind (Hartwig und MAK Commission 2022).

Daher wird Palmkernöl der Schwangerschaftsgruppe C zugeordnet.

**Krebserzeugende Wirkung. **Kanzerogenitätsstudien sind mit Palmkernöl nicht durchgeführt worden. Die Triglyceride Lardöl, Palmöl, Rapsöl, Sojaöl und Kokosnussöl haben kein kanzerogenes Potenzial (Hartwig und MAK Commissi﻿on 2019, 2022). Eine Einstufung in eine Kategorie für krebserzeugende Arbeitsstoffe erfolgt für Palmkernöl daher nicht.

**Keimzellmutagene Wirkung. **Zu Palmkernöl liegen keine Untersuchungen zur Genotoxizität vor. Für die Triglyceride Lardöl, Palmöl, Rapsöl, Sojaöl und Kokosnussöl ist kein genotoxisches Potenzial gegeben (Hartwig und MAK Commission 2019, 2022). Ein Strukturverdacht auf eine genotoxische Wirkung ist für Palmkernöl nicht anzunehmen. Daher wird für den Stoff keine Einstufung in eine Kategorie für Keimzellmutagene vorgenommen.

**Hautresorption. **Zur Hautresorption von Palmkernöl liegen keine quantitativen Daten vor. Modellberechnungen können aufgrund der variablen Zusammensetzung und des extrem hohen log K_OW_ nicht angewendet werden. Eine für Palmkernöl ermittelte orale LD_50_ von > 5000 mg/kg KG und die regelmäßig tolerierte Aufnahme ähnlicher Triglyceride (z. B. Palmöl, Rapsöl, Sojaöl) durch den Menschen im g/kg-KG-Bereich mit der Nahrung deuten auf eine geringe systemische Toxizität dieser Öle hin. Da somit selbst hohe dermale Penetrationsraten keine systemischen Effekte erwarten lassen, unterbleibt eine Markierung von Palmkernöl mit „H“.

**Sensibilisierende Wirkung. **Zur sensibilisierenden Wirkung von Palmkernöl liegen keine Befunde beim Menschen und keine tierexperimentellen Untersuchungen oder Untersuchungen mit tierversuchsfreien Alternativverfahren (NAMs) vor. Palmkernöl wird daher weder mit „Sh“ noch mit „Sa“ markiert.
